# Genetic diversity and the emergence of ethnic groups in Central Asia

**DOI:** 10.1186/1471-2156-10-49

**Published:** 2009-09-01

**Authors:** Evelyne Heyer, Patricia Balaresque, Mark A Jobling, Lluis Quintana-Murci, Raphaelle Chaix, Laure Segurel, Almaz Aldashev, Tanya Hegay

**Affiliations:** 1 Eco-anthropologie et Ethnobiologie, UMR7206 Département Hommes Natures Sociétés, Musée de l'Homme - 17, Place du Trocadéro - 75116 Paris, France; 2 Department of Genetics, University of Leicester, Adrian Building, University Road, Leicester, LE1 7RH, UK; 3 Human Evolutionary Genetics Unit, CNRS URA3012, Institut Pasteur, Paris, France; 4 Institute of Molecular Biology and Medicine, National Center of Cardiology and Internal Medicine, Bishkek, Kyrgyzstan; 5 Uzbek Academy of Sciences, Institute of Immunology, Tashkent, Uzbekistan

## Abstract

**Background:**

In this study, we used genetic data that we collected in Central Asia, in addition to data from the literature, to understand better the origins of Central Asian groups at a fine-grained scale, and to assess how ethnicity influences the shaping of genetic differences in the human species. We assess the levels of genetic differentiation between ethnic groups on one hand and between populations of the same ethnic group on the other hand with mitochondrial and Y-chromosomal data from several populations per ethnic group from the two major linguistic groups in Central Asia.

**Results:**

Our results show that there are more differences between populations of the same ethnic group than between ethnic groups for the Y chromosome, whereas the opposite is observed for mtDNA in the Turkic group. This is not the case for Tajik populations belonging to the Indo-Iranian group where the mtDNA like the Y-chomosomal differentiation is also significant between populations within this ethnic group. Further, the Y-chromosomal analysis of genetic differentiation between populations belonging to the same ethnic group gives some estimation of the minimal age of these ethnic groups. This value is significantly higher than what is known from historical records for two of the groups and lends support to Barth's hypothesis by indicating that ethnicity, at least for these two groups, should be seen as a constructed social system maintaining genetic boundaries with other ethnic groups, rather than the outcome of common genetic ancestry

**Conclusion:**

Our analysis of uniparental markers highlights in Central Asia the differences between Turkic and Indo-Iranian populations in their sex-specific differentiation and shows good congruence with anthropological data.

## Background

Central Asia is located on the Silk Road, where numerous ethnic groups characterised by different languages and historical modes of subsistence co-exist. These include the Tajik populations, who speak an Indo-Iranian language and are sedentary agriculturalists, and several Turkic populations, who speak an Altaic language and are traditionally nomadic herders [1,2]. However, some of the latter (e.g. Uzbeks) have shifted to a sedentary agriculturally-based lifestyle more recently, during the sixteenth century. These two groups of populations have different lifestyles, but also different social organisations. Agriculturalist societies are patrilocal and are organised into families. Marriage rules are based on kinship and geographical proximity with a strong preference for first-cousin marriages. Conversely, nomadic societies are organised into so-called "descent groups", namely "lineages, clans, and tribes". Individuals belonging to each of these descent groups claim to share a recent common ancestor on the paternal line. We have previously shown that such claims have a biological basis for individuals belonging to lineages and clans, but that links between individuals from a given tribe and their claimed paternal ancestor are socially constructed rather than biological [3]. Membership of these descent groups is transmitted through the father to the children, and we have previously shown that the dynamics of these descent groups increase the Y-chromosomal inter-population genetic differentiation among Turkic populations [4], in comparison to the level of Y-chromosomal differentiation among agriculturalist populations and reduces male effective population size [5].

However, the level at which Central Asian groups are genetically differentiated, in particular for the Y chromosome, remains unclear. Indeed, it remains to be understood whether the genetic variation differentiates primarily ethnic groups (e.g. Uzbeks versus Kazakhs, etc.) or whether it differentiates primarily populations within ethnic groups (e.g. Kyrgyz from the lowlands, versus Kyrgyz from the mountains). More generally, the underlying question is whether ethnicity is the major determinant of genetic differences between populations. We are also interested in understanding better the processes leading to the emergence of ethnic groups, and in understanding the extent to which constituted ethnic groups are endogamous. One focus of this study was to assess the levels of genetic differentiation between ethnic groups on one hand and between populations of the same ethnic group on the other hand in order to understand better how ethnicity shapes the genetic diversity of human populations, and to give insights on the processes leading to the formation of ethnic groups. To address this question, we sampled several populations per ethnic group (from 2 to 6 populations per ethnic group) from the two major linguistic groups in Central Asia.

An additional aim of this study was to use genetic data to understand better the history and formation of particular Central Asian ethnic groups. Indeed, parts of their history remain controversial. Among the Turkic groups, the Karakalpaks, Uzbeks and Kazakhs are thought to be subgroups of the same Uzbek confederation that emerged during the fifteenth century following the collapse of the Golden Horde after the dissolution of Genghis Khan's empire. The Karakalpak group emerged more recently and resulted from a split from the Kazakh confederation in the seventeenth century. However, the origin of the Kyrgyz living in Kyrgyzstan is still a matter of debate in the scholarly literature. Late in the eighth century the Kyrgyz state was a major rival of the Great Turkic Empire and later defeated the Uighur in the ninth century. The prevailing current opinion is that part of this Kyrgyz population moved from South Siberia to Kyrgyzstan in the fifteenth century and included some nomadic groups that inhabited the region for several centuries. Turkmen tribal genealogies trace their origin to the Oghuz who lived in the area in the sixth century. The agriculturalist Tajik sedentary populations speak a western Indo-Iranian language that entered the area through the Muslim invasion in the tenth century, and are perhaps descendants of former eastern Indo-Iranian speakers who have lived there for more than two millennia. For all historical references see [1,2]. In this study, we used genetic data that we collected in Central Asia, in addition to data from the literature (24 populations, 846 individuals for mitochondrial DNA and 20 populations, 745 individuals for the Y chromosome), to understand better the origins of Central Asian groups at a fine-grained scale, and to assess how ethnicity influences the shaping of genetic differences in the human species.

## Results

### Mitochondrial DNA variation

We investigated how the genetic variance, based on mtDNA haplotype frequencies (HVS-I sequences) was distributed in a hierarchical mode using an AMOVA analysis [6]. The overall differentiation was low but statistically significant (Fst = 0.013; P < 0.000). Differences among ethnic groups explain about 0.6% (P < 0.001) of the overall variance. The comparison of Turkic populations versus Tajik Indo-Iranian populations showed that differences between these two groups constitutes 0.55% (P < 0.0283) of the total genetic variance. Intra-ethnic group genetic differentiation was significant for the Tajik group (Fst = 0.0197; P < 0.001) but not for the Turkic groups (0.3% P = 0.10). Differences among Turkic ethnic group was low but globally significant (0.66% P < 0.001). [See additional file 1].

TABLE 1

**Table 1:** Intra ethnic-group genetic differentiation based on HVSI.

Language family	Ethnic group	Intra-group differentiation	Probability
Turkic	Karakalpak (N = 3)	0.05%	0.37
Turkic	Kazakh (N = 3)	0.00%	0.58
Turkic	Kyrgyz (N = 6)	0.67%	0.06
Turkic	Turkmen (N = 3)	0.38%	0.238
Turkic	Uzbek (N = 4)	0.19%	0.33
Indo-Iranian	Tajik (N = 5)	**1.97%**	**0.000**

N: number of populations per ethnic group. Probability: the probability that an Fst will be higher than the observed value in 1000 permutations

When taking into account all populations, no correlation between genetic and geographical distances was detected at the global level (Mantel test, r = -0.00682, P = 0.502). This lack of correlation remains if we test separately for each language family.

### Y-chromosomal variation

With respect to the Y chromosome, the AMOVA analysis performed using the 20 populations showed that about 5.6% of genetic differentiation is due to differences among ethnic groups (P < 0.02) and that the overall differentiation between populations is RST = 0.186 (P < 0.001). When populations were grouped by language affiliation/mode of subsistence -- Turkic versus Tajik -- ~9.1% of the genetic variability was due to differences between these two groups. In addition, the analysis at the intra-group level revealed a high degree of differentiation both for Tajik and Turkic populations except for the two Uzbek populations. [See additional file 1].

TABLE 2

**Table 2:** Intra ethnic-group genetic differentiation based on 7 Y-chromosomal microsatellites.

Language family	Ethnic group	Intragroup differentiation: Rst	Probability
Turkic	Karakalpak (N = 2)	9.03	0.000
Turkic	Kazakh (N = 3)	15.6	0.000
Turkic	Kyrgyz (N = 6)	7.35	0.000
Turkic	Turkmen (N = 2)	25.1	0.000
Turkic	Uzbek (N = 2)	0.009	0.21
Indo-Iranian	Tajik (N = 5)	22.94	0.000

N: number of populations per ethnic group. Probability: the probability that an Rst will be higher than the observed value in 1000 permutations

A Mantel test of correlation between geographical and genetic distance was non-significant (r = -0.0145 p = 0.4755). Note: this test was based only on 19 populations since KRI-TY could not be assigned a precise geographical location (individuals were sampled in a military camp and come from several places in Kyrgyzstan). This lack of correlation remains if we test inside language family or even among a sub-region for one ethnic group (Kyrgyz).

TABLE 3

**Table 3:** BATWING results for each ethnic group

	Ne	Alpha	Time of first split (generations)	Time of first split (years)			Historical estimates
Karakalpak	1779 (1128-2797)	0.004 (0.0008-0.0089)	29.3 (12.08-53.13)	878.9 (362.4-1593.93)			400
Kazak	1636 (1036-2585)	0.005 (0.0011-0.0107)	88.59 (45.87-148.13)	2657.61 (1375.98-4444.05)			600
Kyrgyz	2914 (2054-4070)	0.0024 (0.0005-0.0051)	55.26 (27.32-95.16)	1657.75 (819.47-2854.74)			600*
Turkmen	1523 (796-2925)	0.0053 (0.001-0.0119)	50.69 (21.49-94.13)	1520.79 (644.82-2823.99)			1500
Uzbek	14088 (6765-23942)	0.0108 (0.0065-0.0155)	41.09 (7.33-87.79)	1232.71 (219.78-2633.73)			600
Tajiks	6585 (3845-10600)	0.0082 (0.0045-0.0123)	62.61 (33.16-106.73)	1878.23 (994.94-3202)			unknown

Effective population size (Ne), growth rate (a) and Time of the first split. Ne is calculated by dividing θ by twice the mutation rate (0.0021) see [20] and [21]. Confidence Intervals represent respectively the 2.5% and 97.5% proportions of the distribution. Generation time of 30 years [22] The model assumes constant population growth. * this estimate is for Kyrgyz living in Kyrgyzstan

Kyrgyz, Kazak, Turkmen and Karakalpak have significantly lower effective population sizes than Tajik and Uzbek populations. Conversely, Uzbek and Tajik populations show higher growth rates but confidence intervals overlap the growth rates of other populations, except for the Kyrgyz when compared with the Uzbek. The date of the first split event is older than 1000 years except in the case of the Karakalpak, but confidence intervals are large.

## Discussion

In this study we addressed, by analyzing uniparentally-inherited markers, how social organisation in human populations can have an impact on genetic diversity. More specifically, we studied the extent to which the way individuals choose their mates and where they settle affect genetic distances between populations.

In the current study, as expected, the overall levels of genetic differentiation based on mtDNA turned out to be very low (less than 1%), even when comparing population groups with different language family affiliations and diverse modes of subsistence. This lack of differentiation most likely results from high levels of female gene flow in these patrilocal societies. The mean Fst among populations of the same ethnic group clearly shows a contrasting pattern between Turkic versus Tajik populations. Among Turkic groups, Fst based on mtDNA is close to zero in all comparisons (except one case of one Kyrgyz population) in contrast with Tajik farmer populations where Fst between populations is always relatively high (0.025). This reflects a different mode of exchanging spouses between populations, with a high level of exchange in the Turkic group and a lower level in the Tajik group [4].

The situation for the Y chromosome in these populations is in sharp contrast with the mtDNA data. Previous studies have reported the occurrence of high levels of Y-chromosomal genetic diversity in Central Asia [4,7,8]. Our study strengthens these observations and most importantly, shows that genetic differentiation is strong even within a single ethnic group. The level of genetic differentiation is lower at the inter-ethnic group level than at the intra-ethnic group level: 5.6% of the differences are among ethnic groups while the overall genetic differences are 18.6%, leaving 13.7% of differences among populations within ethnic group. The differences among populations belonging to the same ethnic group vary according to the ethnic group with a non significant value for the two Uzbek populations, a lower value for Karakalpak and Kyrgyz (7% and 9% respectively) and a higher value for Turkmen (25%). This observation cannot be accounted by the geographic location of these populations since there is no global correlation between genetic and geographical distances, nor a physical barrier between them.

### Contrasting levels of differentiation for Y and mtDNA

We found evidence that overall, the Y chromosome has a significantly higher level of differentiation between populations than does mtDNA, in agreement with previous studies. The present study also shows that the level at which differentiation occurs is different between the two markers. There are more differences between populations of the same ethnic group than between ethnic groups for the Y chromosome, whereas the opposite is observed for mtDNA in the Turkic group. Indeed, no differences are observed in the Turkic group between populations belonging to the same ethnic group but there is a significant (although low) genetic differentiation between ethnic groups. This is not the case for Tajik populations where the mtDNA differentiation, like Y-chromosomal differentiation, is also significant between populations within this ethnic group.

**Figure 1:**
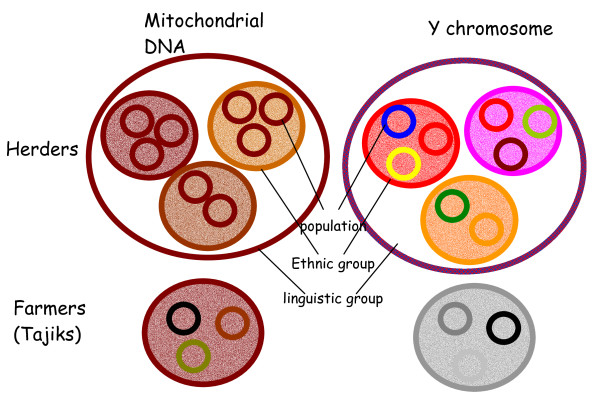
**Schematic representation of genetic structuring at the ethnic group level**. The different colours indicate genetic differences, with shades of a particular colour indicating relatively small differences. Each small circle represents a population, intermediate circles stand for an ethnic group.

Ethnologists describe the social organisation of Turkic populations as exogamous at the clan level or the lineage level (depending on the population) but endogamous at the tribe level - a man chooses his spouse outside the clan or his lineage but inside the tribe and inside his ethnic group. The geographical spread of a given tribe is wide [1], and this could explain the lack of mtDNA genetic differentiation between populations that are defined on geographical criteria. However, we would have expected stronger differences between ethnic groups. One explanation for our observations of low levels of maternal differentiation could be that ethnic groups are not actually highly endogamous. An ethnological study during our field expedition in Karakalpakia measured the level of endogamous mating at the tribe level among Karakalpak. Of 506 matings considered, 443 (87.5%) were among members of the Karakalpak ethnic group, and 78.5% among members of the same tribe [9]. Thus, even if the ethnic group's endogamy is high (87.5%) from an ethnological perspective, it is low from a genetic point of view and insufficient to create high levels of genetic differentiation for mtDNA between these ethnic groups. An alternative explanation is that ethnic groups are a recent aggregation of tribes of different origins. This low level of endogamy, combined with an aggregation of unrelated tribes to form an ethnic group, leads to a low level of matrilineal genetic differentiation among ethnic groups. By contrast, Tajik populations are endogamous - a male tends to choose his spouse in the same village, and within the same family. This is shown by the significant Fst between Tajik populations for mtDNA. Further, the strong sex-specific difference in the pattern of genetic differentiation in Turkic populations (i.e. no mtDNA genetic differences between populations but strong Y-chromosomal differences within them) is explained by their strongly patrilineal social organisation. This type of organisation is absent in Tajik and explains the less sex-specificity in the genetic differentiation observed in this ethnic group (see Figure 1), consistent with no sex-specificity in the effective population size that has been demonstrated recently [5].

### History of ethnic groups

The combination of mtDNA and Y-chromosomal data from these large collections of populations and ethnic groups of Central Asia can shed light on the history of these groups. In addition, the Y-chromosomal analysis of genetic differentiation between populations belonging to the same ethnic group can give some estimation of the minimal age of these ethnic groups. The median estimate of the age of first split is always older than 1000 years (except for Karakalpak, for which it is 880 years). Actually, this estimation does not represent the age of the group *sensu stricto*, but the lower bound at which the group originated. In any case, this estimate is older than what is known from historical records for most of the Turkic ethnic groups, further, even if the confidence intervals are large, they do not overlap with historical estimates in two of the ethnic groups (and marginally three). Historical sources state that the Kazakh, Kyrgyz and Uzbek living in Central Asia arose in the sixteenth century. Genetic data show that populations belonging to one of these ethnic groups have an older common ancestor (more than one thousand years ago). Although these estimates are based on only one genetic system (linked Y chromosome microsatellites), we can propose that these ethnic groups are a heterogeneous conglomerate of tribes or populations. This hypothesis has been previously formulated in the case of Brahmin caste in India, whose subcastes seem to result from a fusion rather than a fission process [10]. Such heterogeneous conglomerate of populations could have its origins at the foundation of the ethnic group or later during its history, as a result of the agglomeration of new unrelated tribes. The second hypothesis is compatible with historical records regarding the Uzbek and the Kyrgyz. Soucek [11] records that what is now called 'Uzbek' encompasses the seventeenth century Uzbek and former Chagatai Turk groups who were already settled in Uzbekistan. Therefore the name refers to a tribal union of different tribes including Chagatai Turks who were strongly mixed with Iranian dwellers of Central Asia. The same type of scenario is proposed by historians regarding the Kyrgyz living in Kyrgyzstan: this group is made up of Kyrgyz who arrived in the country in the fourteenth century and of Turkic groups who were already leaving in TienShan. The minimum age of the origin of the group is compatible with a common ancestry for the Turkmen group. This does not prove the common ancestry hypothesis, but does not refute it formally as for the other ethnic groups. In any case, additional sampling would certainly help to test these hypotheses, especially because our Turkmen group is composed of only two populations. Similar analyses based on mtDNA information are not feasible because of the high uncertainty in mtDNA mutation rate calibration and the near absence of genetic differentiation among populations belonging to the same ethnic group. Recent common ancestry or older common ancestry with high levels of gene flow are both possible explanations for this absence of mtDNA genetic differentiation. Despite the limitations associated with mtDNA data, our study shows that for the Turkic, there is a slight but significant mtDNA genetic differentiation between ethnic groups. This is consistent with the results on the Y chromosome revealing genetic differentiation between ethnic groups. The refutation of the common ancestry hypothesis for several of these ethnic groups, together with the observation of inter-group genetic differentiation, suggest that genetic boundaries separate them.

## Conclusion

Since the work of Frederik Barth in the 1970s [12] anthropologists have placed emphasis not only on presumed common ancestry and shared cultural traits, but also on the "boundaries" used by individuals in order to distinguish themselves from members of other ethnic groups. These boundaries can take different forms - racial, cultural, linguistic, economic, religious, and political - and may be more or less porous. The persistence of such boundaries implies rules. One of the most common rules around the world is an endogamous preference for mate choice. In conclusion, our analysis of uniparental markers lends support to Barth's hypothesis by indicating that ethnicity, at least for two (and marginally three) of the Turkic groups in Central Asia, should be seen as a constructed social system maintaining genetic boundaries with other ethnic groups rather than the outcome of common genetic ancestry. It further highlights the differences between Turkic and Indo-Iranian populations in their sex-specific differentiation and shows good congruence with anthropological data.

## Methods

### Samples

**Figure 2:**
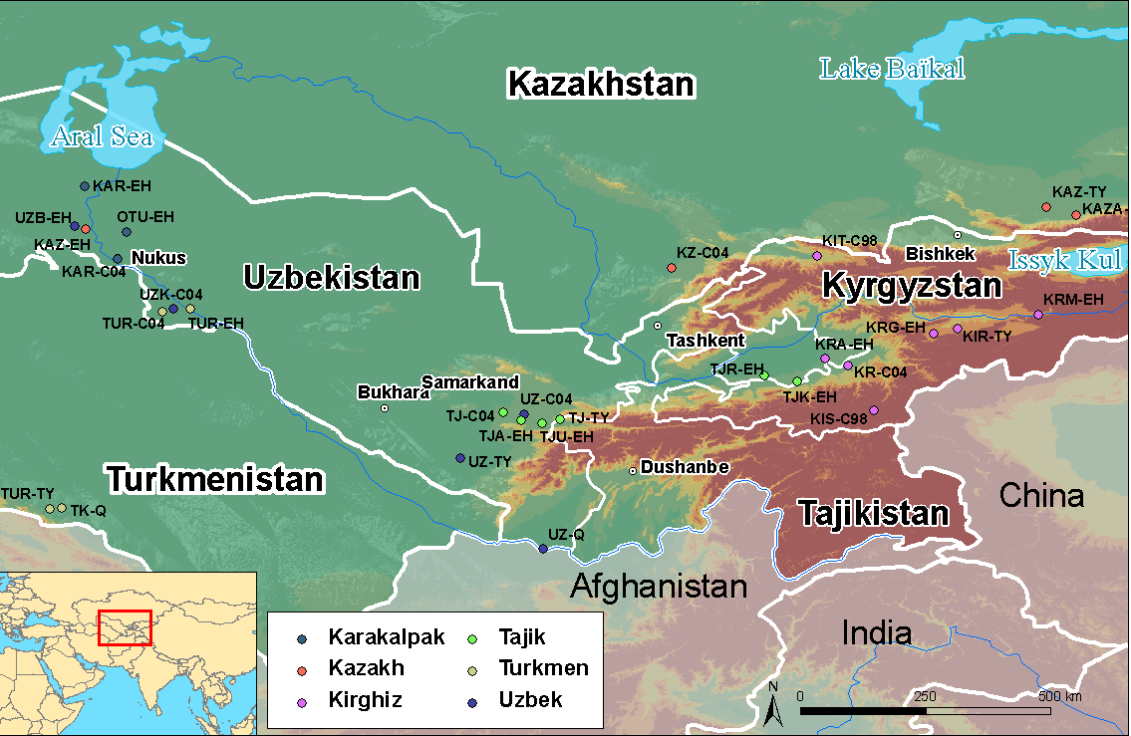
**Geographic map of the sampled area**.

We combined our results with previous data published on the populations of Central Asia - [see additional file 2] for the list of populations and Figure 2 for their locations [4,7,13-15]. For each village, our sampling strategy was to sample individuals who were more distantly related than the first and second degree, and belonged to the same ethnic group. Such samples are considered as "populations" in our study. Regarding data from the literature, the sampling strategy is not always precisely described and when the information was not published, we contacted authors to obtain more detailed information. Except for one Kyrgyz sample (not included in the geographical analyses), all samples followed the strategy adopted by us.

### Molecular Methods

DNA was extracted from blood samples using standard protocols. Informed consent was obtained from all participants.

#### Mitchondrial DNA

The first hypervariable segment (HVS-I) of the control region was sequenced in all samples, and variable positions were determined from position 16024 to 16383, as previously described [14]. The C-tract length variation at positions 16182 and 16183 in HVS-I was excluded from the analysis. Sequence quality was ensured as follows: each base pair was determined once with a forward and once with a reverse primer; any ambiguous base call was checked by additional and independent PCR and sequencing reactions; all sequences were examined by two independent investigators.

#### Y Chromosome

Y chromosome diversity was assessed using a set of microsatellites, since these are variable in all populations and avoid the possible ascertainment bias associated with Y-SNPs. We typed 12 microsatellites on the Y chromosome, but for comparison with previous studies, we present the result for only seven of these. According to the protocol described by [16], we genotyped and analysed the microsatellites DYS388, DYS389I, DYS392, DYS19, DYS390, DYS391 and DYS393.

### Statistical Analysis

In order to determine how overall genetic diversity is distributed within and between populations, an analysis of molecular variance (AMOVA) was performed using Arlequin v 2.0 software [6]. For mtDNA, the mutation model assumed was the Kimura 2-parameter model with a transition/tranversion ratio of 10 and an alpha (Gamma shape parameter) of 0.26. For the Y-linked microsatellites, we used the RST genetic distance [17], which takes into account the probability of recurrent mutation. We performed a global AMOVA analysis including all populations and also considering several groupings corresponding to the ethnic affiliation of populations. For the ethnic grouping, we divided populations into six ethnic groups: Karakalpak, Kazakh, Uzbek, Kyrgyz, Turkmen and Tajik. Correlations between genetic and geographical distances were performed using a Mantel test implemented in the R package [18].

Based on the generally high levels of population differentiation observed with the Y-chromosomal microsatellites we decided to perform a BATWING [19] analysis to estimate different population history parameters: (a) the population parameter θ for the populations altogether of the same ethnic group (2Mu, where u is the mutation rate [20,21]and M is equal to Ne - the effective size - for a uniparentally inherited gene and to 2Ne for a biparentally inherited gene); (b) the total growth rate; (c) the parameters of the population 'supertree', namely the dates of the splitting events, the identity of the populations that split and the proportional size taken up by each population.

The program assumes that the populations under study have diverged from an ancestral population at different points in time, have the same growth rate (growth or stationarity can be assumed) and have not exchanged migrants after the splits. The date of the first split represents the minimum age of the ethnic group. A generational interval of 30 years was assumed [22].

## Authors' contributions

EH conceived of the study and participated in its design and coordination, performed the statistical analysis, collected the Central Asian samples, drafted the manuscript, PB carried out the molecular genetic studies, participated in the design of the study and helped to draft the manuscript, MAJ participated in the design of the study and helped to draft the manuscript, LQ participated in the design of the study and helped to draft the manuscript, RC participated in the design of the study, participated in the collection of samples and helped to draft the manuscript, LS participated in the design of the study and helped to draft the manuscript, AA participated in the design of the study, participated in the collection of samples and helped to draft the manuscript, TH participated in the design of the study, participated in the collection of samples and helped to draft the manuscript. All authors read and approved the final manuscript.

## Supplementary Material

Additional file 1 **Amova analysis and MDS representation of mitochondrial and Y chromosome genetic distances among populations**. Click here for file

Additional file 2 **List of samples**. IE Indo-European language, TK Turkic language. 1: [15], 2: [13], 3: [7], 4: [14], 5: [4], 6: Present study. Click here for file
