# Supplementary material for: Genetic diversity and the emergence of ethnic groups in Central Asia
Source: BMC Genet. 2009 Sep 1;10:49. doi: 10.1186/1471-2156-10-49 (PMC2745423; doi:10.1186/1471-2156-10-49)
Supplement: Additional file 1 — Amova analysis and MDS representation of mitochondrial and Y chromosome genetic distances among populations. [file 1471-2156-10-49-S1.doc]

|  | d.f. | Sum of Squares | Percentage of variation |
| --- | --- | --- | --- |
| Among groups: TK versus Tajik | 1 | 9.2 | **0.55** |
| among populations within Tajik | 4 | 19.1 | **1.97** |
| Within Tajik populations | 145 | 434 | 98.03 |
|  |  |  |  |
| Among TK groups | 4 | 28.1 | **0.66** |
| Among populations within TK groups | 14 | 52.8 | 0.30 |
| among population within UZB | 3 | 9.5 | 0.19 |
| among population within TUR | 2 | 6.7 | 0.38 |
| among populations within KIR | 5 | 16.4 | 0.67 |
| among populations within KAZ | 2 | 5.9 | 0 |
| among populationswithin KAR | 2 | 6.7 | 0.05 |
|  |  |  |  |
| Within TK populations | 677 | 2313 | 99.03 |
|  |  |  |  |
| Total | 845 | 2848.6 |  |

Table S1 : AMOVA mtDNA 24 Populations

|  | d.f. | Sum of Squares | Percentage of variation |
| --- | --- | --- | --- |
| Among groups: TK versus Tajik | 1 | 192.757 | **9.11** |
| Among populations within Tajik | 4 | 262.966 | **22.94** |
| Within Tajik populations | 142 | 961.864 | **77.06** |
|  |  |  |  |
| Among TK groups | 4 | 248.236 | **4.90** |
| among population within UZB | 1 | 10.882 | 0.88 |
| among population within TUR | 1 | 49.567 | **25.16** |
| among populations within KIR | 5 | 87.123 | **7.35** |
| among populations within KAZ | 2 | 54.817 | **15.65** |
| among populationswithin KAR | 1 | 47.824 | **9.03** |
|  |  |  |  |
| Within TK populations | 583 | 3063.904 | 86.39 |
|  |  |  |  |
| Total | 744 | 4979.94 |  |

Table S2 : AMOVA Y-chromosome 7 microsatelites 20 Populations


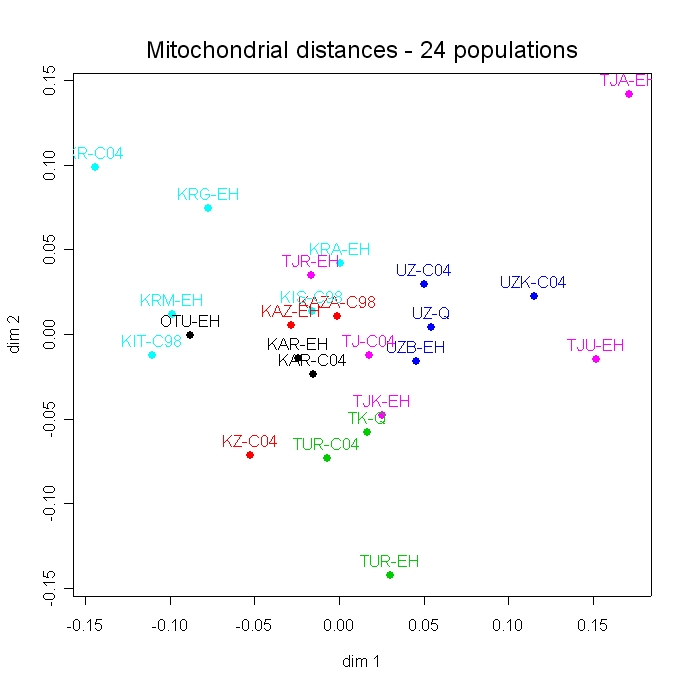


Figure S1 : MDS for mitochondrial distances


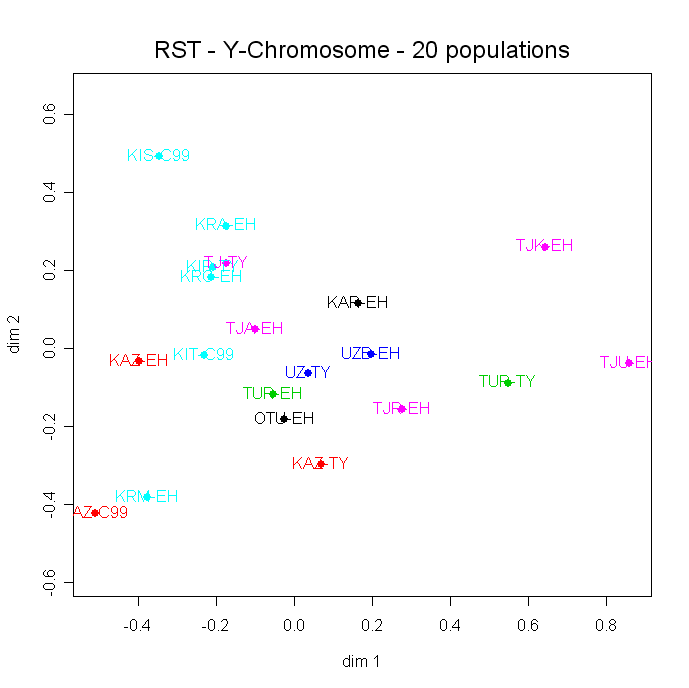


Figure S2 : MDS for Y-chromosome distances
