# Supplementary material for: Genetic diversity and the emergence of ethnic groups in Central Asia
Source: BMC Genet. 2009 Sep 1;10:49. doi: 10.1186/1471-2156-10-49 (PMC2745423; doi:10.1186/1471-2156-10-49)
Supplement: Additional file 2 — List of samples. IE Indo-European language, TK Turkic language. 1: [15], 2: [13], 3: [7], 4: [14], 5: [4], 6: Present study. [file 1471-2156-10-49-S2.pdf]

Ethnic group	Population	Population Code	Langage affiliation	Way of life	mtDNA N	Y chromosome N	Ref.
Karakalpak	Nukus, Uzbekistan	KAR-C04	TK	Nomadic	20		2
Karakalpak	Qongrat from Karakalpakia ; Uzbekistan	KAR-EH	TK	Nomadic	55	54	5
Karakalpak	On Tört Uruw from Karakalpakia ; Uzbekistan	OTU-EH	TK	Nomadic	53	54	5
Kazakh	Almaty, Katon-Karagay, Karatutuk, Rachmanovsky Kluchi ; Kazakstan	KAZ-TY	TK	Nomadic 1-2 generations ago		38	1
Kazakh	Kazak Collected in Gazli, Uzbekistan, but most originally from region of Chimkent, Kazakstan (1-2 generations ago)	KZ-C04	TK	Nomadic 1-2 generations ago	20		2
Kazakh	Aktasty, Almaty region ; Kazakhstan	KAZ-C99 / KAZA-C98	TK	Nomadic 1-2 generations ago	55	49	3
Kazakh	Karakalpakia; Uzbekistan	KAZ-EH	TK	Nomadic 1-2 generations ago	50	50	5
Kyrgyz	central Kyrgyzstan (mixed) ; Kyrgyzstan	KIR-TY	TK	Nomadic		41	1
Kyrgyz	Osh area, Kyrgyzstan	KR-C04	TK	Nomadic	20		2
Kyrgyz	Alai Valley; Kyrgyzstan	KIS-C99	TK	Nomadic	47	43	3
Kyrgyz	Talas Valley - Kyrgyzstan	KIT-C99	TK	Nomadic	48	41	3
Kyrgyz	Fergana Valley, Andijan area; Uzbekistan	KRA-EH	TK	Nomadic	48	46	5
Kyrgyz	Lower Naryn district ; Kyrgyzstan	KRG-EH	TK	Nomadic	20	20	5
Kyrgyz	Upper- Naryn district; Kyrgyzstan	KRM-EH	TK	Nomadic	26	22	5
Uzbek	Kashkadarya area ; Uzbekistan	UZ-TY	TK	Recent		28	1

				agriculturalist (XVth)			
Uzbek	Surkhandaria area; Uzbekistan	UZ-Q	TK	Recent agriculturalist (XVth)	42		4
Uzbek	Urgench area; Uzbekistan	UZK-C04	TK	Recent agriculturalist (XVth)	20		2
Uzbek	Samarkand area, Uzbekistan	UZ-C04	TK	Recent agriculturalist (XVth)	20		2
Uzbek	Karakalpakia; Uzbekistan	UZB-EH	TK	Recent Agriculturalist (XVth)	40	40	5
Turkmen	Ashgabat ; Turkmenistan	TUR-TY	TK	Nomadic		21	1
Turkmen	Urgench, Uzbekistan	TUR-C04	TK	Nomadic	20		2
Turkmen	Ashgabat ; Turkmenistan	TK-Q	TK	Nomadic	41		4
Turkmen	Karakalpakia; Uzbekistan	TUR-EH	TK	Nomadic	51	51	5
Tajik	Penjikent ; Tajikistan	TJ-TY	IE	Agriculturalist		22	1
Tajik	Samarkand; Uzbekistan	TJ-C04	IE	Agriculturalist	20		2
Tajik	South Ferghana valley; Uzbekistan	TJK-EH	IE	Agriculturalist	40	35	6
Tajik	West Ferghana valley; Uzbekistan	TJR-EH	IE	Agriculturalist	29	29	6
Tajik	Urgut area ; Uzbekistan	TJU-EH	IE	Agriculturalist	29	29	6
Tajik	Samarkand area; Uzbekistan	TJA-EH	IE	Agriculturalist	32	32	6
